# Rapid Gelling of Guar Gum Hydrogel Stabilized by Copper Hydroxide Nanoclusters for Efficient Removal of Heavy Metal and Supercapacitors

**DOI:** 10.3389/fchem.2021.794755

**Published:** 2021-11-16

**Authors:** Xinwei Zhu, Yingxi Chen, Renjian Xie, Haijian Zhong, Weidong Zhao, Yang Liu, Hui Yang

**Affiliations:** ^1^ Key Laboratory of Prevention and Treatment of Cardiovascular and Cerebrovascular Diseases, Ministry of Education, Gannan Medical University, Ganzhou, China; ^2^ Key Laboratory of Biomaterials and Bio-fabrication in Tissue Engineering of Jiangxi Province, Ganzhou, China; ^3^ School of Rehabilitation Medicine, Gannan Medical University, Ganzhou, China; ^4^ First Affiliated Hospital of Gannan Medical University, Ganzhou, China; ^5^ School of Medical Information Engineering, Gannan Medical University, Ganzhou, China; ^6^ Institute of Biomedical Engineering and Health Sciences, Changzhou University, Changzhou, China

**Keywords:** guar gum, polysaccharides, hydrogel, copper hydroxide, crosslinking

## Abstract

In this paper, guar gum (GG) hydrogel has been successfully prepared by adding GG and Cu^2+^ mixture into an alkaline medium. The formation mechanism of the hydrogel has been investigated through various techniques. Results reveal GG facilitates the formation of ultrafine copper hydroxide clusters with a diameter of ∼3 nm. Moreover, these nanoclusters bring about a rapid gelling of GG within 10 ms. The synthesized hydrogel is applied to the adsorption of heavy metal ions from wastewater. The hydrogel shows excellent removal efficiency in removing various heavy metal ions. Besides, the hydrogel derived porous carbon exhibits high specific capacitance (281 F/g at 1 A/g) and excellent rate capacity. The high contaminant removal efficiency character and excellent electrochemical performance endow GG hydrogel with potential applications in the environmental and energy storage field.

## Introduction

The acceleration of industrialization has aggravated water quality deterioration and energy depletion, which is becoming a worldwide concern. Heavy metal ions are one of the main components of industrial effluents, and the issues are attracting intense attention from the research and management community (Y. Wu et al., 2019). It has been recognized that heavy metal ions always give rise to highly toxic even at an extremely low concentration (Tchounwou et al., 2012). Besides, electrochemical energy storage system is thought to be a promising technology for replacing fossil fuels. Hence, over recent decades, researchers have focused extensively on developing advanced materials for eliminating pollutant and enhancing energy storage/conversion (Geissen et al., 2015).

Up to now, various strategies for removing heavy metals from wastewater have been proposed, such as chemical precipitation (Chen et al., 2018), flocculation (Sun et al., 2020), adsorption (Saya et al., 2021a), electrochemical techniques (Hu et al., 2020), etc. While the following drawbacks hinder these approaches: high cost, time-consuming processing, inefficient for treating pollutants at a low level, etc. Chemical precipitation with the addition of alkaline is a common strategy for wastewater treatment in the traditional industry. However, amorphous metal hydroxides will be partially dissolved in an alkaline environment, so chemical precipitation cannot remove heavy metal ions wholly. Moreover, the effective separation of these fine precipitates from water is still challenging. Compared with other methods, adsorption is one of the most promising techniques for treating heavy metal ions because of its eco-friendly, low cost, simplicity, and high-efficiency characteristics. Till now, numerous adsorbent materials (Hasanpour and Hatami, 2020), including carbon-based materials, metal-organic framework, and other porous materials, have been prepared. The industrial application prospects of these materials are still restricted by several aspects, such as complex preparation process, low output, high cost, etc.

Natural polysaccharides hydrogels are thought to be promising candidates of adsorbents owing to their non-toxicity, naturally abundant, tunable structures, and composition features (Aminu et al., 2019; Qi et al., 2018). Moreover, polysaccharides hydrogel-derived carbon materials are also ideal candidate precursors of 3D graphitic porous carbon owing high specific surface area, which is vital for enhancing the electrochemical performance of electrode materials. Guar gum (GG) is a typical complex polysaccharide obtained from the seed of the GG plant. Generally, natural gums tend to form 3D interconnected networks known as a hydrogel, while the hydrogel is viscoelastic fluids and unable to maintain a stable structure (Anandha Kumar and Sujatha, 2021). So far, there are many reports of GG-based hydrogel fabricated by crosslinking with inorganic/organic crosslinkers (Cheng et al., 2020; Dinari et al., 2020). Nevertheless, the slow gelling process and low productivity of these crosslinking agents make them hard to satisfy the practical application demand. In our previous study, GG/Cu^2+^ mixture contacted an alkaline solution resulted in a rapid gelling phenomenon (Hui et al., 2019). Similar phenomenon has been reported in other polymer systems. Liu reported dextrin coprecipitated with metal hydroxide but not with metal ions in an alkaline medium through a chemical complex mechanism (Liu and Laskowski, 1989). Furthermore, Yokoi found copper hydroxide-like nanoclusters complexed with poly (vinyl alcohol) driven by hydrophobic interaction (Hiroshi et al., 1995). Till now, the information related to GG hydrogel stabilized by copper ions under alkaline medium is limited, and the corresponding regulation factors and gelation mechanism are still unclear.

Herein, a facile method with rapid gelling and high-yield characteristics has been proposed for preparing GG hydrogel. The GG-based hydrogel is obtained by adding the mixture containing GG and Cu^2+^ into an alkaline medium, which results in the formation of GG hydrogel within a short time. In addition, the corresponding hydrogel is adjustable in shape from sphere, fiber, and membrane by altering the mixing mode. The forming process of the hydrogel is detected with the aid of a high-speed video camera. The hydrogel has been investigated by SEM, TEM, EDS, FTIR, XRD, XPS, and viscosity testing to investigate the GG and copper hydroxide interaction and unveil the gelation mechanism. Furthermore, the potential applications of the GG-based hydrogel for supercapacitor and adsorption of heavy metal ions have also been explored.

## Materials and Methods

### Materials

Copper nitrate trihydrate (Cu(NO_3_)_2_·3H_2_O, Analytically Pure (AR)), iron nitrate nonahydrate (Fe(NO_3_)_2_·9H_2_O, AR), cobalt nitrate hexahydrate (Co.(NO_3_)_2_·6H_2_O, AR), nickel nitrate hexahydrate (Ni(NO_3_)_2_·6H_2_O, AR), GG (5,000–5,500 cps, 200 meshes) with a molecular weight of ∼1.7 × 10^6^ g/mol (Li et al., 2018), potassium hydroxide (KOH, AR), hydrochloric acid (HCl, AR, 36%–38%) were all purchased from Shanghai Aladdin Bio-Chem Technology Co., LTD (China). These agents were used directly without further purification unless otherwise specified.

### Preparation of Cu/GG Hydrogel

In this paper, GG-based hydrogels with different morphology, including spheres, fibers, and membrane, have been prepared. For the synthesis of Cu/GG spheres, 0.25 g GG and 0.1208 g Cu(NO_3_)_2_·3H_2_O were dissolved into 50 ml deionized water. After stirring for 4 h at room temperature, the colloid mixture was added dropwise into KOH solution (1M, 50 ml), hydrogel spheres were formed immediately, and these spheres were labeled as Cu/GG spheres. Cu/GG fiber was synthesized by injecting the mixture containing GG and Cu^2+^ into KOH solution by a syringe with the needle size of 0.24–0.7 mm, and the corresponding jet speed was 0.5 ml/s. In addition, Cu/GG membrane has been prepared according to the following procedure. The mixture containing Cu^2+^ and GG was transferred into a plastic culture dish, and KOH solution (1M, 50 ml) was added. The light-green hydrogel was dried at 50°C for 6 h. After that, the membrane was stripped from the dish with the aid of a blade, and immersed in DI water for 30 min to remove the redundant KOH. The final membrane was obtained through natural drying.

### Characterization

The shape transformation of the hydrogel sphere was observed by a high-speed video camera (V211-80, Phantom, United States) at a frame rate of 2000 frames/s. Typically, to increase the contrast between hydrogel spheres and the background, 0.5 ml black ink was added into the GG/Cu colloidal solution (50 ml). GG/Cu/ink colloidal mixture was then added into KOH solution (1 M) dropwise by a burette. A high-speed camera recorded the motion of liquid drop, and all experiments were conducted at room temperature.

The surface morphology of the samples was studied by scanning electron microscopy (SEM, SIGMA, NETZCH, Germany) with an accelerating voltage of 5 kV. The equipment was equipped with energy dispersive X-ray spectrometry (EDS, Ultim Max, Oxford, United Kingdom), which was applied to analyze the elemental distribution of the samples. The transmission electron microscopy (TEM) images were recorded on Tecnai F20 (FEI, United States) with an EDS detector. The diameter of the hydrogels was obtained using Nano Measurement software (Nanomeasurer 1.2.5), and the statistics were analyzed by SPSS 25.0 software (SPSS, United States). The products’ crystal phase and chemical composition were characterized by XRD (X'Pert PRO MPD, Panalytical, Holland). The operating voltage and current were 40 kV and 40 mA, respectively. The scanning scope was from 10^o^ to 80^o^ at a scanning rate of 2 ^o^/min. The chemical state of the products was tested by using an X-ray microprobe (ESCALAB 250XI, Thermo, United States) equipped with Al Kα radiation. The chemical composition of the samples was recorded by FTIR testing on Nicolet IS 10 (Thermo, United States). The spectra of the samples ranging from 4,000 to 400 cm^−1^ were obtained, and their corresponding scanning rate was 2 cm^−1^. The viscosity data of the hydrogels were obtained by viscometer (DV2T, Brookfield, United States) at a shear rate of 200 1/s.

The adsorption performance of the hydrogel for heavy metal ions was evaluated, and Cu^2+^, Co^2+^, Ni^2^+, Mn^2+^, and Pb^2+^ were chosen as the models. Typically, the pH of GG solution (1 ml, 0.5 wt%) was adjusted to 12 by KOH solution, and the mixture was poured into the solution containing heavy metal ions (50 ml, 0.1 mg/ml). Then, a hydrogel formed and precipitated at the bottom of the mixture. After soaking for 5 min, the concentration of residual ions was determined by Inductively coupled plasma mass spectrometry (ICP-MS, Perkin Elmer, NexION350). The removal effciency of the ions was determined by Equation (1).

$$removal\;efficiency\;=\frac{C_{0}-C_{e}}{C_{e}}\times 100\%$$ (1)

Where C_0_ and C_e_ were initial and equilibrium concentrations of ions (mg/L), respectively. The stability of these hydrogels was evaluated by prolonging the soaking period. A certain volume of supernatant was collected at regular time intervals, and the concentration of these samples was measured by ICP-MS to monitor the dissolution behavior of the hydrogel. Besides, the potential effect of the pH on the hydrogel adsorption behavior was also investigated.

The electrochemical performance of the hydrogel-derived porous carbon has been evaluated by a three-electrode system with the aid of CHI 760 electrochemical workstation. Typically, Cu/GG spheres were dried at 60 °C for 12 h. The precursor was carbonized at certain elevated temperatures (400, 600, 800 and 900°C) for 2 h under Ar atmosphere, and washed with deionized. These samples are labelled as HPCS-400, HPCS-600, HPCS-800 and HPCS-900, respectively. After that, a slurry containing porous carbon, acetylene black and polytetrafluoroethylene was obtained and coated onto a graphite paper, which was dried and used as the working electrode. Besides, the Ag/AgCl electrode and Pt were applied as the reference electrode and counter electrode respectively. Cyclic voltammetry (CV) and Galvanostatic charge/discharge (GCD) measurements were elevated with a voltage range of −1–0 V. The electrochemical impedance spectroscopy (EIS) spectra of the HPCSs was tested at an current amplitude of 5 mV within a frequency range of 0.01 Hz–100 kHz. In addition, the cycling stability of the electrode has also been investigated.

## Results and Discussion

**FIGURE 1:**
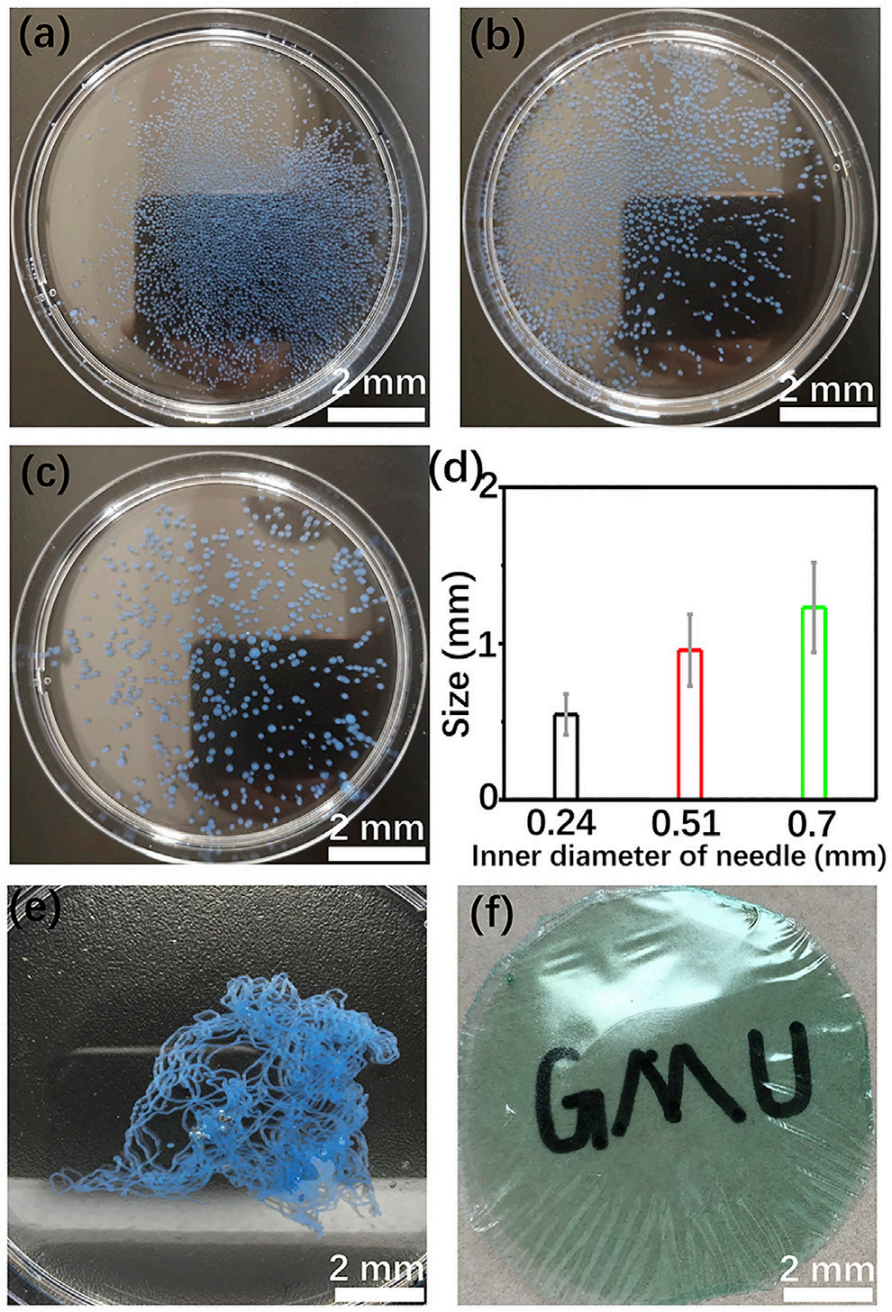
Cu/GG spheres prepared with different sizes of the syringe needle **(A–C)**; **(D)** size distribution of these hydrogel spheres; **(E)** Cu/GG fiber; **(F)** Cu/GG membrane.

The shape and size distribution of the hydrogel has been adjusted by changing the injection mode of GG/Cu^2+^ colloid solution and the diameter of the syringe needle, respectively. Adding the GG/Cu^2+^ colloid solution dropwise into an alkaline environment brings about the formation of spherical hydrogel spheres (Figures 1A–C). The size of the spheres is proportional to the inner diameter of the needles. Raising the needle size from 0.24 to 0.7 mm, the average diameter of the spheres transforms from 0.54 to 1.23 mm, respectively (Figure 1D). After soaking in deionized water for over 7 days, part of these hydrogel spheres will be collapse because of swelling, and the pH of the medium increases to 8.3, indicating a small amount of basic matter encapsulated in the hydrogel. Enhancing the concentration of Cu^2+^ (100 mM) is beneficial to stabilize the hydrogel structure and inhibit its structure disintegration (Supplementary Figure S1). Hence, the copper compound might perform the role of crosslinking agent. Generally, enhancing the concentration of crosslinking agents hinders the swelling behavior of hydrogel because of a higher crosslinking density, and it further ensures the structural stability of the hydrogel (Lei and Clark, 2007). Rapid injection of the colloid into the alkaline medium leads to the generation of the fibrous hydrogel (Figure 1E). The possibility of building a membrane-based structure is explored. As shown in Figure 1F, flexible GG membranes with good transparency have been obtained. The composite membrane might open a new avenue for organic catalysts, energy storage, and flexible electronic device applications. What’s more, other transition metal ions, including Co^2+^, Ni^2+^, Fe^3+^, also give rise to a rapid gelling process of GG and promote the shaping of GG-based hydrogels (Supplementary Figure S2). Hence, all these transition metal ions might play a similar role in promoting the gelation of GG.

**FIGURE 2:**
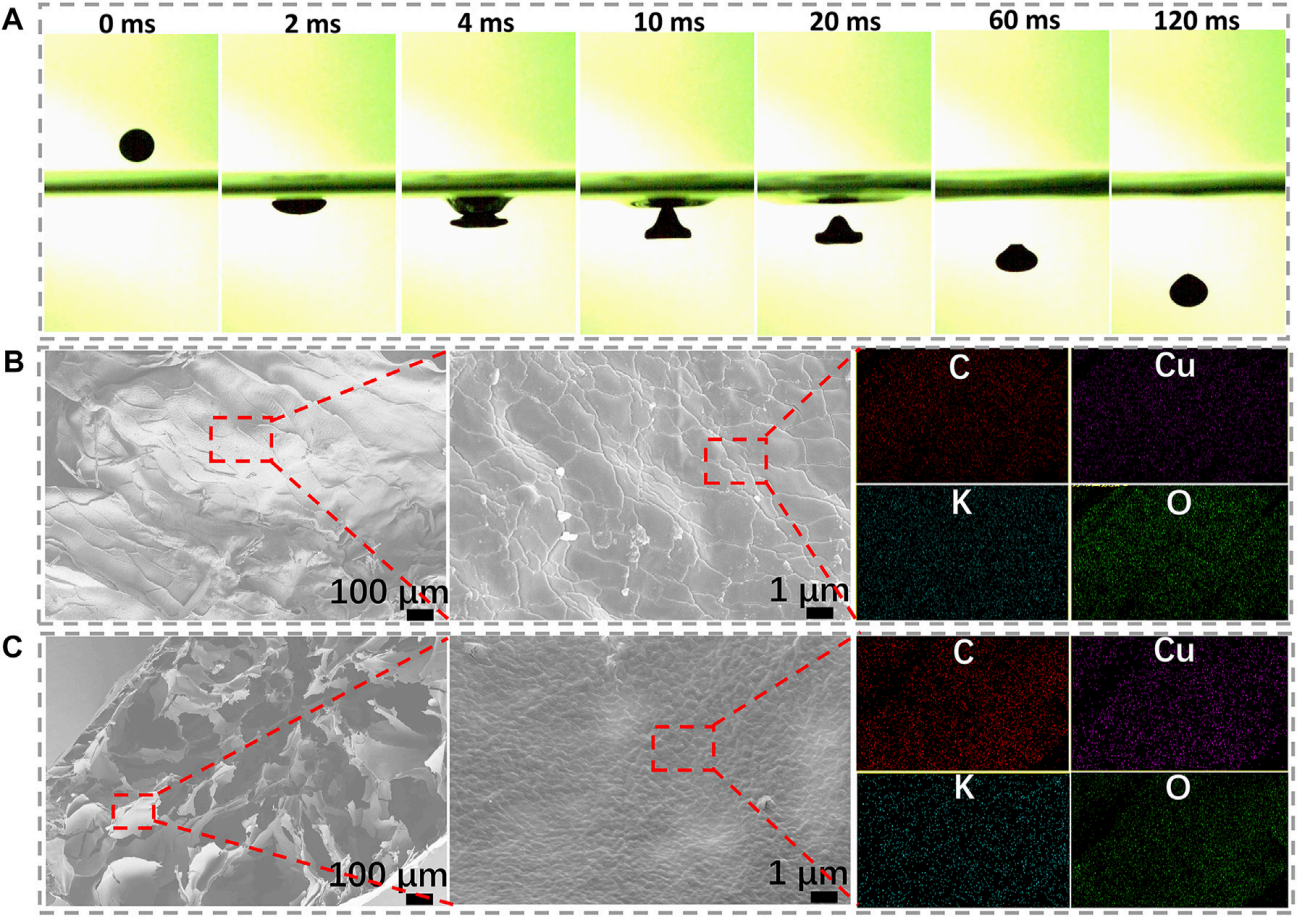
**(A)** High-speed photography images of the hydrogel sphere; **(B)** Surface microstructure and EDS mapping of the hydrogel; **(C)** Cross-section images and EDS mapping of the hydrogel.

A high-speed video camera is applied to record the shape transformation process of spheres. When the spherical Cu/GG liquid drop collides with the surface of the KOH solution, it flattens on the bottom while still maintains an integrality structure (Figure 2A). As the liquid drop immerses into the solution, a notable peaked-cap structure is observed at ∼10 ms. Subsequently, the height of the cap gradually decreases during the following 50 ms, and near-spherical particles form within 120 ms. It is noteworthy that the rapid gelling process ensures the dispersion of spheres, and these hydrogel spheres will not cluster with each other, even raise the dropping rate (Supplementary Movie S2).

Hence, a dense and rigid surface structure should be formed once Cu/GG drop contacts with KOH solution. Because of the unique surface structure, the drop maintains its integrality structure and avoids clustering together (Nayak and Lyon, 2004). The SEM results confirm the hypothesis (Figure 2B). The coarse morphology should be ascribed to the partial shrinkage during lyophilization treatment. EDS mapping results reveal copper element is uniformly distributed onto the sphere shell of Cu/GG. In addition, the cross-section images show a loose network consists of micro-sheets (Figure 2C). Similarly, the surface structure and composition of the sheets are the same as that of the shell (Supplementary Table.S1). Moreover, the porous core structure demonstrates that many gases and liquid components still preserve inside the hydrogel. Further prolonging the soaking period for 24 h, the porous structure still exists (Supplementary Figure S3). Therefore, the preservation of porous core structure can be interpreted as being attributed to the high viscosity of the colloidal solution, which hinders the mass transfer between the medium and the hydrogel. At the same time, it guarantees abundant pore-forming additives, such as water and gases, exist inside the hydrogel (Ji et al., 2011).

Generally, stable hydrogel forms when the polymer molecules strongly interact with the crosslinking agent (Hennink and van Nostrum, 2012; Hoffman, 2012). Hence, to illustrate the actual agent that regulates gelling reaction, the viscosity transitions of GG solution affected by different additives are investigated. As shown in Supplementary Figure S4, at the same shear rate (200 1/s), the GG solution owns a viscosity of 138 cP, which matches the results reported by Subramani (Anandha Kumar and Sujatha, 2021). Because of the nonionic and uncharged characters, GG is stable in a wide range of pH, and its viscosity is not sensitive to the pH value (Sujatha et al., 2020). However, the addition of KOH results in a viscosity decreasing of GG solution, that’s consistent with the results released by Goycoolea, who found alkaline medium reduced the intrinsic viscosity of GG via decreasing molecular weight through degradation (Goycoolea et al., 1995). In addition, copper ions interact with *cis*-hydroxyl groups of GG chains, and slightly increases the colloidal viscosityl (Saya et al., 2021b). Among them, the Cu/GG group exhibits the highest viscosity compared with other groups. Hence, according to the results mentioned above, copper hydroxide should be the real crosslinking agent that governs the formation of Cu/GG hydrogel.

**FIGURE 3:**
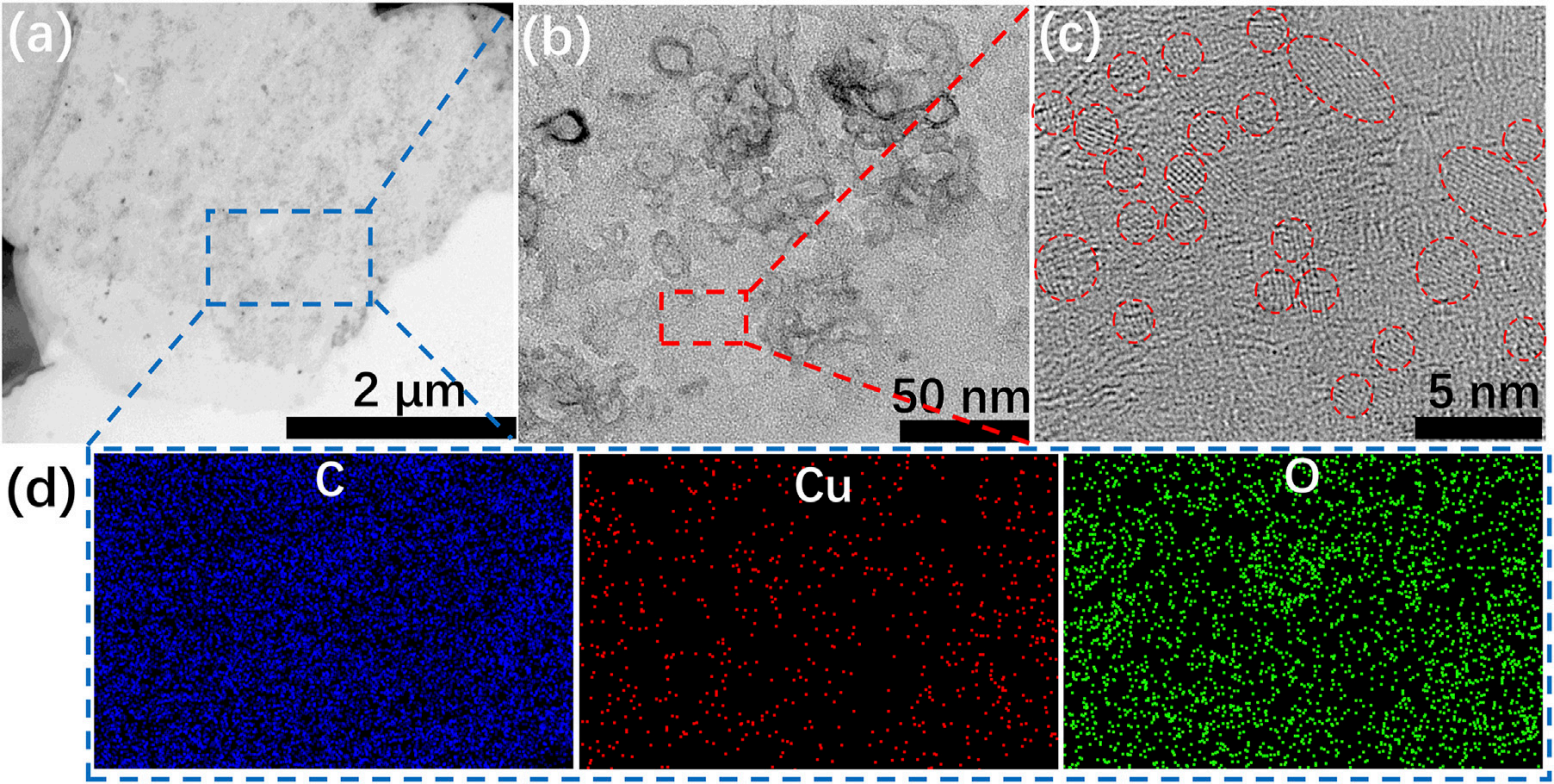
**(A–C)** TEM images of Cu/GG hydrogel; **(D)** elemental mapping images of the selected area labeled in **(A)**.

It is noteworthy that, different mixing order determines the nucleation and growth environment of crystals (Aastuen et al., 1986; De Yoreo and Vekilov, 2003), and then governs the size distribution, crystallinity and shape of copper hydroxide nanocrystals. These crystals act as a crosslinking agent that will further affect the status of the hydrogel. The spherical or fiber-like structure of Cu/GG can only be prepared according to the following procedure: GG mixes with Cu^2+^, then the mixture is added into KOH solution by dropwise or injection. Therefore, the shape and size transition of Cu(OH)_2_ synthesized in different conditions are tested (Supplementary Methods). Directly mixing KOH and Cu(NO_3_)_2_ gives rise to the generation of plate-like Cu(OH)_2_ with a diameter of ∼1 μm (Supplementary Figure S5). The addition of GG in the reaction medium results in the formation of Cu/GG hydrogel with a smooth surface (Figures 3A–C). Besides, the size of copper hydroxide (∼2 nm) dramatically decreases, and the shape of these nanoparticles is spherical (Figure 3E). Moreover, the uniform distribution of the copper element indicates copper hydroxide nanoparticles are tightly confined in the GG matrix (Figure 3F). The XRD results reveal that GG molecular significantly hinders the crystallinity of copper hydroxide (Supplementary Figure S6). During the biomineralization process, polymers (Shao et al., 2018), (Jabalera et al., 2019), (Yao et al., 2020) act as hard templates and regulate the growth and nucleation of minerals through a complicated process, then delicate hierarchical structures assembled by ultra-small particles generate. As a natural polysaccharide, GG can also interact with copper hydroxide nanocrystals, and decrease the crystallinity of the crystals by inhibiting their aggregation and growth tendency. Moreover, high viscosity environment is another crucial factor for reducing the diameter of nanoparticles (Nakashima et al., 2016).

**FIGURE 4:**
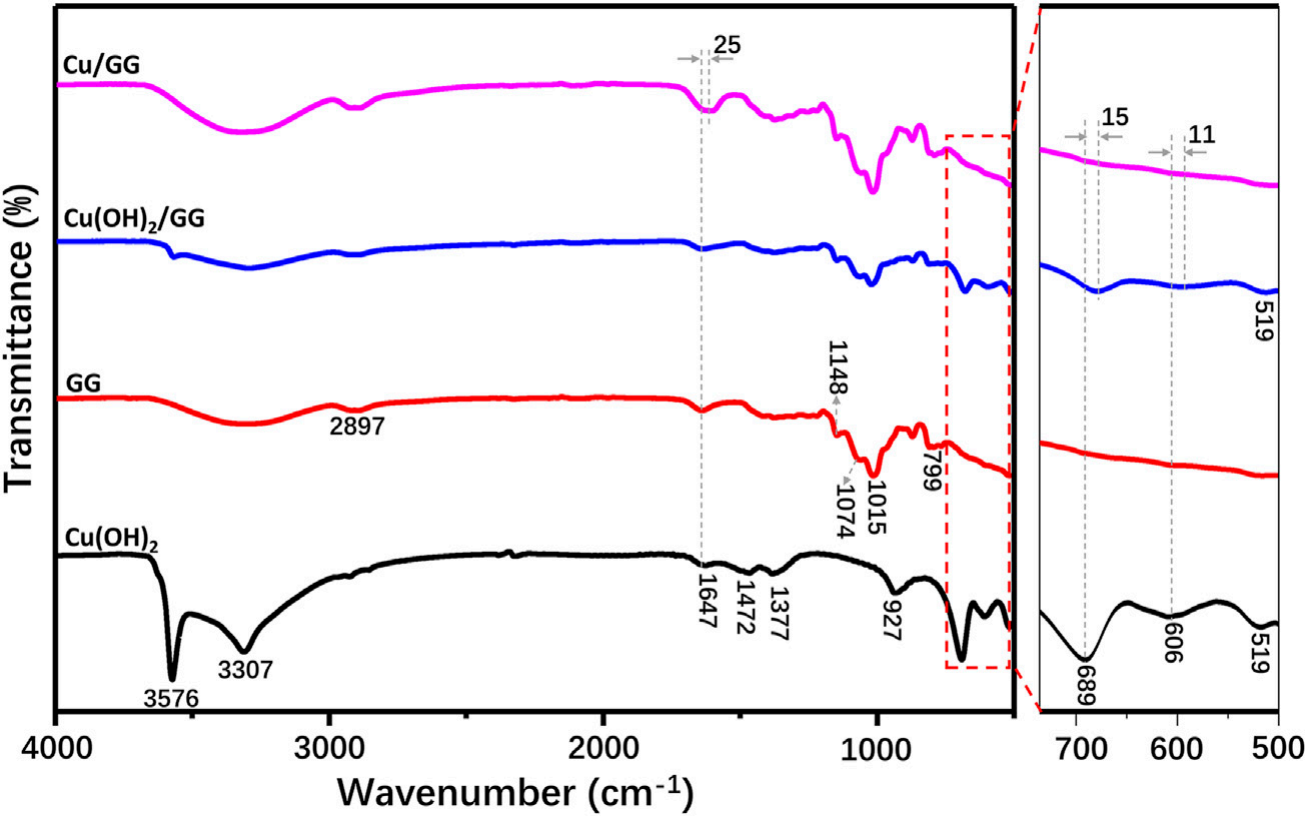
FTIR spectroscopy of Cu/GG, Cu(OH)_2_/GG, GG and Cu(OH)_2_.

FTIR measurement has been applied to verify the strong affinity between copper hydroxide and GG molecules. As shown in Figure 4, FTIR spectra of Cu(OH)_2_, GG, Cu(OH)_2_/GG composite and Cu/GG hydrogel are recorded. For Cu(OH)_2_ group, the bands at 3,576 and 3,307 cm^−1^ are ascribed to the hydrogen-bonded hydroxyl groups. Both peaks at 1,472 and 1,377 cm^−1^ are attributed to hydrogen bonding. The adsorption bands at 927, 606 and 519 cm^−1^ are due to Cu-O stretching bonding, and the band at 689 cm^−1^ is due to the bending vibration of hydrogen-bonded OH groups originated from Cu-O-H bending (Behnoudnia and Dehghani, 2013; Sun et al., 2012). For GG, the bands at 1,148 and 1,074 cm^−1^ are ascribed to the stretching mode of C-OH and CH_2_OH, and the adsorption peak at 1,016 and 2,897 cm^−1^ are attributed to -CH_2_ twisting and stretching vibration, respectively (Chauhan et al., 2009). Note that the band at 1,647 cm^−1^ is due to O-H bending derived from the deformation of the glucose ring (Mudgil et al., 2012), and the corresponding band shifts to 1,622 cm^−1^ in the spectrum of Cu/GG. However, for Cu(OH)_2_/GG hydrogel, a red-shifting phenomenon has not been found. The redshift of the hydroxyl group represents a change from a weak to a strong hydrogen bond and illustrates an enthalpy increase. Compared with larger Cu(OH)_2_ particles, the ultrafine copper hydroxide may serve as a temporary energy donor, which activates and improves the reactivity of hydroxyl groups, and then facilitates the efficient binding interaction of copper hydroxide with the hydroxyl groups on GG (Garczarek and Gerwert, 2006). Moreover, the viscosity testing results reveal the binding strength between GG and Cu(OH)_2_ is more robust than that of GG and CuO (Supplementary Figure S6), indicating hydroxyl groups play an essential role during the gelling process. According to the results of the swelling behavior of Cu/GG, it can be concluded that increasing the content of Cu^2+^ is helpful to enhance the stability of Cu/GG hydrogel (Supplementary Figure S7).

Generally, it is considered that the interaction between metal hydroxide and hydroxyl groups from polymer might be driven by the following process: coordination, complexation, electrostatic, hydrogen bonding, etc.(Zhu et al., 2017). Liu et al. reported that metal hydroxide complexed with the glucose ring through a chemical complexation mechanism, leading to the status changing of the glucose ring (Liu and Laskowski, 1989). The characteristic bands, attributed to Cu-O vibration at 3,576 cm^−1^ and in the range of 500–700 cm^−1^ in the spectrum of Cu(OH)_2_/GG composite, are weaker than that of pure Cu(OH)_2_, suggesting GG hinders the Cu-O vibration by hydrogen bonding or Van der Waal’s force (Maier et al., 1993). However, all these peaks are absent in the Cu/GG hydrogel (Figure 4). So, GG might induce the transformation of the valence state of Cu and modifies the Cu-O stretch mode and status.

**FIGURE 5:**
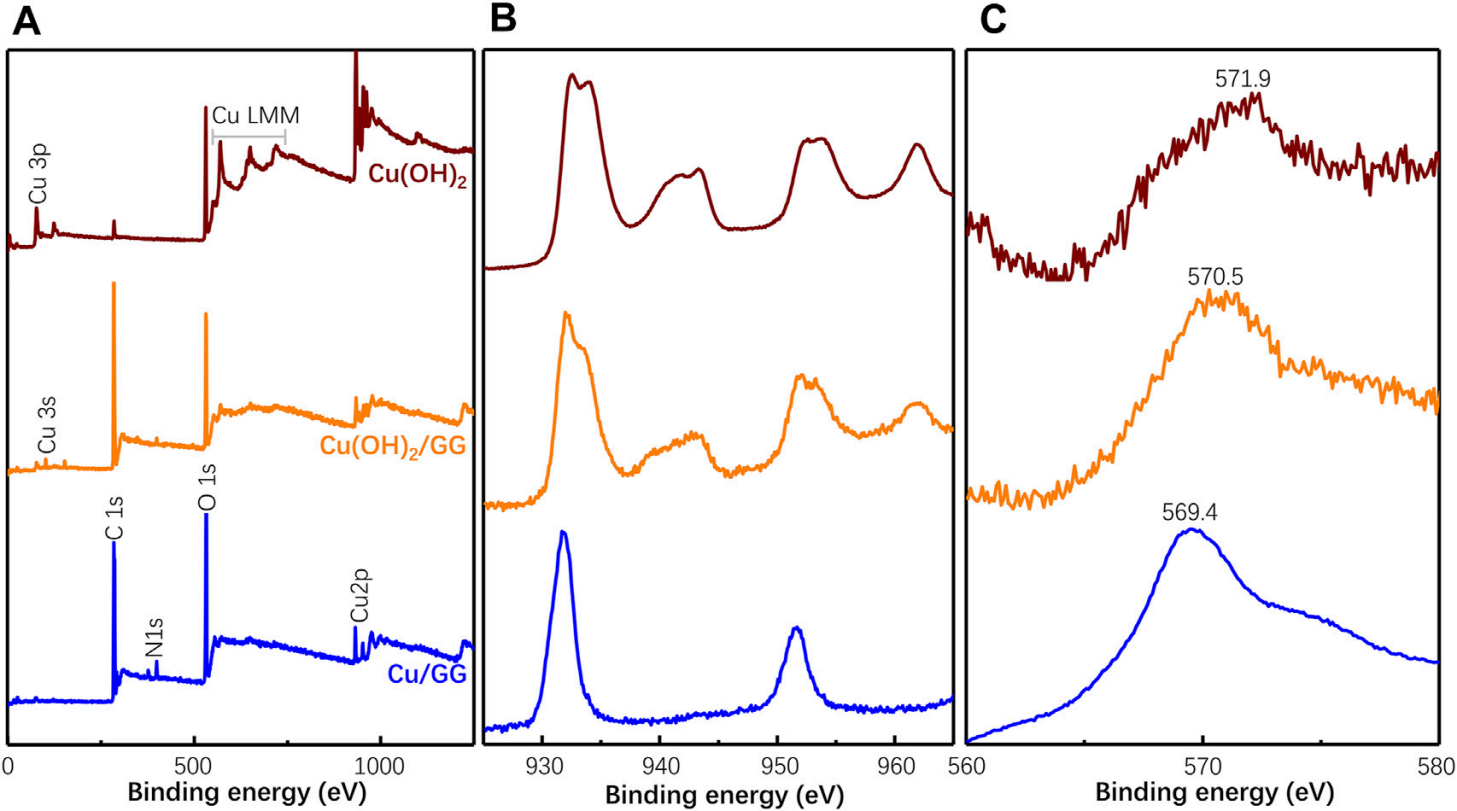
XPS survey of Cu/GG, Cu(OH)_2_/GG and Cu(OH)_2_ **(A)**; the high-resolution of Cu 2p **(B)** and Cu LMM Auger electron **(C)** XPS spectra of the as-obtained samples.

The status of the copper hydroxide cluster is studied through XPS. As shown in Figure 5, based on the survey scan, the two prominent peaks at ∼932 and 953 eV should be attributed to Cu2p_3/2_ and Cu2p_1/2_, respectively. It has been reported that the binding energy of Cu2p in Cu(OH)_2_ and/or CuO is 934.2 eV (Akhavan et al., 2011). As the component peak shifts to 932 eV, a portion of Cu should be in a state rich in electrons. That is attributed to the complexation reaction between copper hydroxide and hydroxy moieties of GG, which donates an electron to the copper center (Fu et al., 2002). Based on the undetectable shift in other samples, the Cu(OH)_2_ microparticles should be weakly complexed with GG molecules. Moreover, the prominent satellite structure at the high binding energy side in identifying Cu(II) compound is missing in the survey scan of Cu/GG. These satellites are assigned to shake-up transitions by ligand  → metal 3 days charge transfer, while the corresponding charge transfer cannot happen in Cu(I) compound because of its complete-filled 3 days shells (Fleisch and Mains, 1982). It has been reported that precious metal ions are supposed to be reduced by terminal alcoholic groups of GG, which finally turned into aldehydic/ketonic groups (Emam et al., 2017; Van Rie and Thielemans, 2017). Furthermore, it has been reported that copper ions boost the oxidation process of hydroxyl groups from polysaccharides and lead to the formation of aldehyde groups (Zhou et al., 2016). Therefore, partially aldehyde groups in the GG structure further reducing Cu(II) to Cu(I) (Wu et al., 2010), which’s consistent with the redox mechanism between GG and copper hydroxide. Therefore, these facts and references suggest that GG reduces Cu(II) hydroxide nanoclusters into Cu(I) compound, or even metallic Cu. Because of the similarity of core level and valence band between metallic Cu, Cu(I), and Cu(II), Cu LMM Auger electrons, which are more sensitive than the Cu 2p core peak, have been measured. As shown in Figure 5C, mixing GG with Cu(OH)_2_ microparticles leads to the peak position shifting from 571.9 to 570.5 eV. In the case of Cu/GG, the corresponding peak further decreases to 569.4 eV, a value close to Cu_2_O (Fleisch and Mains, 1982). Briefly, XPS results reveal GG partially reduces copper hydroxide, and decreasing the diameter of copper hydroxide nanoparticles facilitates the acceleration of the reduction reaction.

It can be concluded the hydrophobic-like character of copper hydroxide nanoparticles boost alkyl chains of GG reacts with copper hydroxide by hydrophobic interaction, then promotes the entanglement of GG chains around the surface of nanoparticles (Hiroshi et al., 1995). The hypothesis is consistent with the polymer protecting mechanism proposed by Naoki et al., who prepared Cu/Pd cluster through PVA-protection effect (Toshima and Wang, 1994). In brief, GG inhibits the growth of copper hydroxide by strong complexation and space confinement effect, resulting in the amorphous copper hydroxide cluster formation. Furthermore, the hydration (Marsh et al., 2014) and complexation (Hiroshi et al., 1995) tendency of clusters significantly influence their geometry and vibration mode of radicals.

**FIGURE 6:**
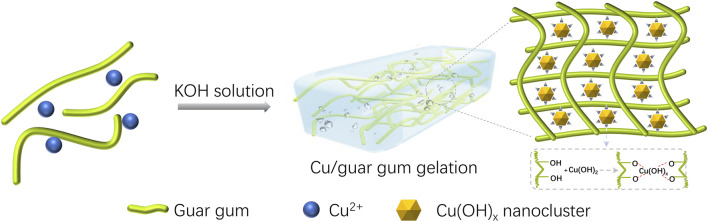
schematic illustration of the rapid gelling process of Cu/GG hydrogel.

Combining the above evidence, a possible shape transformation mechanism is proposed (Figure 6). GG complexed with copper hydroxide results in the generation of ultrafine copper hydroxide nanoparticles with strong reactivity and hydrophobic characters. The copper hydroxide tends to interact with GG to lower its surface energy and balance the entropy loss. It facilitates the entanglement of GG chains around nanoparticles, which will further increase the viscosity of the colloid. These interactions ensure the formation of a rigid surface shell structure at the initial gelling stage. The as-formed dense shell structure plays the following three roles: 1) keeping the structural stability of colloidal drops; 2) inhibiting the aggregation of the hydrogel spheres; 3) lowering the mass transfer between core and medium, then slowing down the gelling rate of the core. The relatively slower gelling process of the core gives rise to a soft inner structure, where contains rich gases and a liquid component. These gases, confined into the high viscosity of GG colloid during the stirring process, provides a slight positive pressure and facilitates spherical particles’ formation even after deformation because of collision.

**FIGURE 7:**
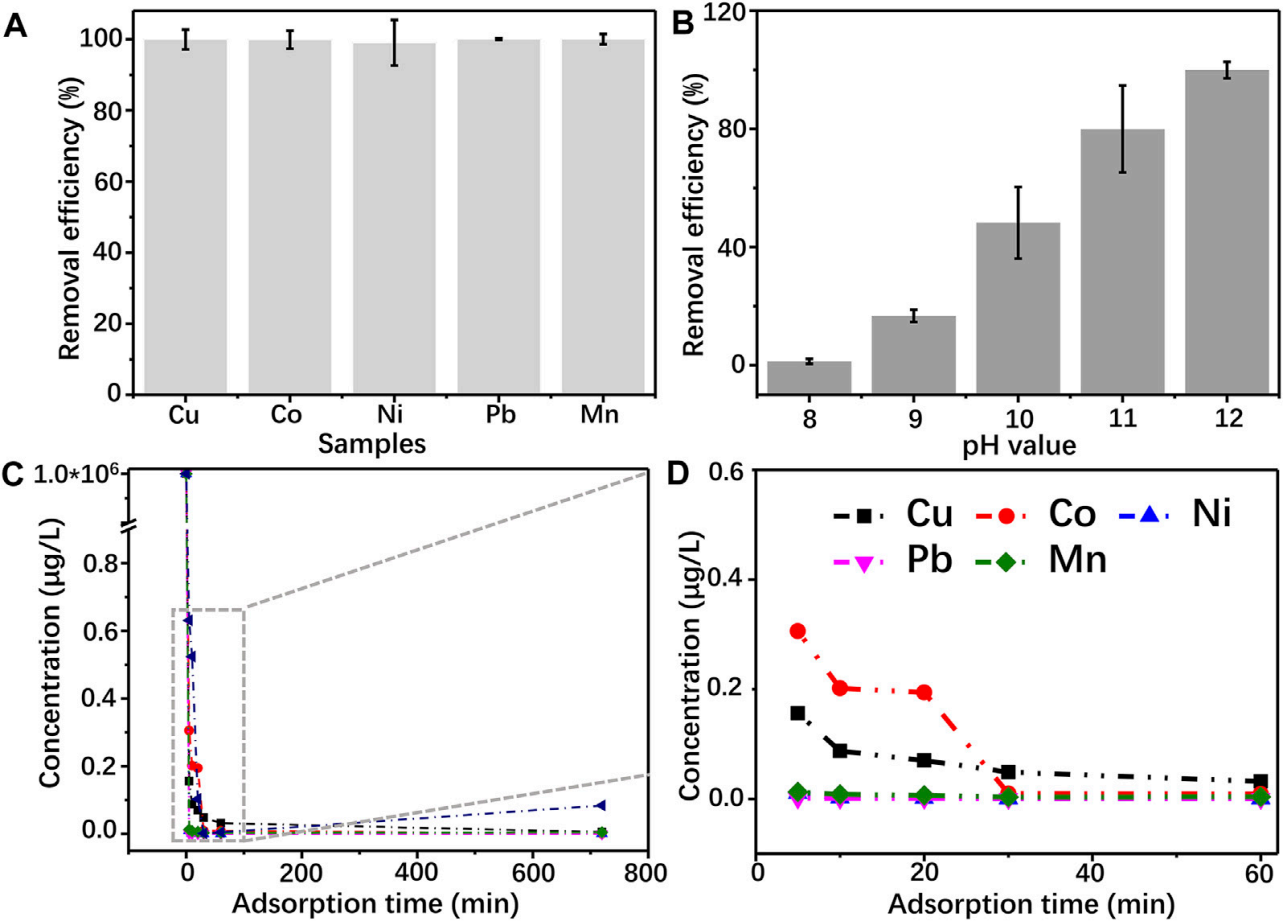
**(A)** removal efficency of Cu^2+^, Co^2+^, Ni^2+^, Pb^2+^, and Mn^2+^on GG hydrogel; **(B)** effect of pH on the adsorption of Cu^2+^ by GG hydrogel **(C, D)** the stability testing of the GG hydrogel after adsorbing heavy metal ions.

In practical application, there are several kinds of heavy metal ions that exist in wastewater. So, it is necessary to require a suitable adsorbent that owns good adsorption behavior to various ions. Herein, to evaluate the adsorption performance of GG hydrogel, several typical heavy metal ions, including Cu^2+^, Co^2+^, Ni^2+^, Pb^2+^, and Mn^2+^, are selected as the research objects. The GG hydrogel shows an excellent removing efficiency in treating these ions, which indicates GG owns a high affinity with metal hydroxide (Figure 7A). Generally, the adsorption of metal ions by hydrogel is driven by the following interactions, such as electrostatic interaction, chelation, ions exchange, etc (Wang et al., 2021). Studies reveal that hydroxyl and amino groups with electron-rich character are prone to bind with metal ions and promote the ions removal efficiency of the hydrogel (Xu et al., 2015). The conclusion is consistent with the FTIR results, which show an apparent redshift of the hydroxyl group (Figure 4). Furthermore, the high affinity between GG and Cu(OH)_2_ results in a partial reduction of Cu^2+^ to Cu^+^ (Figure 5), indicating the adsorption should be through physical and chemical adsorption.

As one of the essential parameters influencing the adsorption capacity of adsorbents, the pH value of the solution determines the ionization state of the adsorbent molecule and further alters the affinity between adsorbed matters and sorbents (Yan et al., 2011; Jiang et al., 2019). Hence, Cu^2+^ is chosen as a model to evaluate the adsorption capacity of the hydrogel (Figure 7B). The removal efficiency of Cu^2+^ is proportional to the pH value of the medium. As the initial pH value is lower than 9, GG shows an extremely low adsorption capacity of Cu^2+^. Further enhancing the pH to 10, 11 and 12, the corresponding removal efficiency of Cu^2+^ is 48.21, 79.96 and 99.91%, respectively. These results are consistent with the results above. Moreover, the stability of Cu/GG, Co./GG, Ni/GG, Pb/GG and Mn/GG hydrogels formed under pH = 12 is evaluated. As shown in Figures 7C,D, the ions concentration drastically decreases from 100 mg/L to lower than 0.5 μg/L within 5 min. Moreover, no apparent ions releasing phenomenon happens after prolonging the immersion time, indicating the high stability of these hydrogels. This method can quickly remove the metal ions in the solution in a short time, and the colloid product can be separated from the water body by simple filtration, so it is a suitable candidate of adsorbent for treating large quantities of wastewater.

**FIGURE 8:**
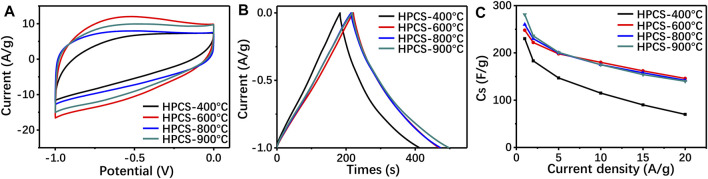
the CV curves (100 mV/s) **(A)**, GCD curves (1 A/g) **(B)** and specific capacitance of the Cu/GG derived porous carbon with different concentration of Cu^2+^; the CV curves (100 mV/s) (d), GCD (e) curves (1 A/g) and specific capacitance of Cu/GG hydrogel derived carbon prepared under different carbonization temperature.

Generally, carbonization temperature alters the microstructure of porous carbon, and further affects the number of active sites of the electrode materials. The pore structure of the final products is listed in Supplementary Table.S2, and results reveal that the carbonization temperature is proportional to the specific surface area of carbon. Moreover, the carbonization also determines the degree of crystallinity of the porous carbon, which is a vital parameter for determining the electrical conductivity of the materials (Li et al., 2015). Therefore, the potential application of the Cu/GG-derived porous carbon as supercapacitor materials has been evaluated, and the potential effects caused by the carbonization temperature has also been investigated. The microstructure measurement results reveal that all samples exhibit porous structure, and the porosity of the carbon is proportional to the carbonization temperature (Supplementary Figure S9). Moreover, as shown in Figure 8, there is no obvious faradaic redox peaks can be observed, and the rectangular shaped cyclic voltammetry curves indicating that the carbon materials are promising electronic double layer capacitor. The copper nanoparticles are stable enough even under high scanning rate condition (Supplementary Figure S10), that should be attributed to protection effect of carbon shell. Furthermore, the GCD results reveal that the enhancement of the carbonization temperature is beneficial to the improvement of the electrochemical performance. However, further increasing the carbonization temperature from 600 to 900°C, although the graphitization degree of the carbon was slightly increased (Supplementary Figure S11), the specific capacitance of the porous carbon exhibits no obvious difference (Figure 8B). According to the electrochemical impedance spectroscopy (EIS) results (Supplementary Figure S12), all samples exhibit an ultralow equivalent series resistance. So, we conclude the copper nanoparticles embedded into the carbon matrix facilitate the electron transfer during electrochemical reaction process. Besides, the rate performance of Cu/GG derived porous carbon has been assessed (Figure 8C), results reveal HPCS-900 owns the most excellent performance, and the corresponding capacitance of HPCS-900 is 281, 236, 201, 175 and 140 F/g at discharge current density of 1, 2, 5, 10 and 20 A/g, respectively, which is better than that of other polyssacharide-derived porous carbon materials (Cui et al., 2018). Furthermore, the cycling charge/discharge of the HPCS has been evaluated at a discharge current density of 10 A/g, the specific capacitance of the electrode materials still maintains 97.4% after 5,000 cycles (Supplementary Figure S11). Taking together, the Cu/GG derived porous carbon is a promising electrode material for supercapacitor.

## Conclusion

In summary, a facile synthetic strategy has been proposed to construct Cu/GG hydrogel with different shapes. Results demonstrate copper hydroxide is a sufficient crosslinking agent for GG. The stability of the hydrogel is inversely proportional to the diameter of copper hydroxide particles. GG inhibits the aggregation of copper hydroxide and guarantees the formation of ultrafine copper hydroxide. Meanwhile, copper hydroxide nanoclusters promote a rapid gelling of GG within 10 ms. The GG-based hydrogel exhibits excellent adsorption performance on various heavy metal ios, and Cu/GG hydrogel derived porous carbon achieves high specific capacitance (281 F/g at 1 A/g) and excellent rate capability. Taken together, the Cu/GG hydrogel is a promising candidate of adsorbents for wastewater treatment and precursor of porous carbon materials for high-performance supercapacitor.

## Declaration of Competing Interest

We declare that we have no financial and personal relationships with other people or organizations that can inappropriately influence our work. There is no professional or other personal interest of any nature or kind in any product, service and/or company that could be construed as influencing the position presented in, or the review of, the manuscript entitled.

## Data Availability

The original contributions presented in the study are included in the article/Supplementary Material, further inquiries can be directed to the corresponding authors.
