# Supplementary material for: Rapid Gelling of Guar Gum Hydrogel Stabilized by Copper Hydroxide Nanoclusters for Efficient Removal of Heavy Metal and Supercapacitors
Source: Front Chem. 2021 Nov 16;9:794755. doi: 10.3389/fchem.2021.794755 (PMC8637109; doi:10.3389/fchem.2021.794755)
Supplement: Supplementary file 1 [file DataSheet1.PDF]

Supporting information

For

Rapid gelling of guar gum hydrogel stabilized by copper hydroxide nanoclusters for efficient removal of heavy metal and supercapacitors

Zhu Xinwei[1,2,3], Chen Yingxi[5], Xie Renjian[1,2,4], Zhong Haijian[1,2,4], Zhao Weidong[1,2,4], Liu Yang[6*], Yang Hui[1,2,4*]

[1]Key Laboratory of Prevention and treatment of cardiovascular and cerebrovascular diseases, Ministry of Education, Gannan Medical University, Ganzhou, China

[2]Key Laboratory of biomaterials and bio-fabrication in tissue engineering of Jiangxi Province, Ganzhou, China.

[3]School of Rehabilitation Medicine, Gannan Medical University, Ganzhou, China.

[4]School of medical information engineering, Gannan Medical University, Ganzhou, China.

[5]First Affiliated Hospital of Gannan medical University, Ganzhou, China.

[6]Institute of Biomedical Engineering and Health Sciences, Changzhou University, Changzhou, China

[*]Corresponding author, E-mail address: liuyang@cczu.edu.cn; yanghui_2521@163.com.

Contents

Supplementary materials......3
Methods......3
Figure s1 (a) size distribution of Co/GG, Cu/GG, Fe/GG and Ni/GG beads. (b) size distribution of Co/GG, Cu/GG, Fe/GG and Ni/GG fibers ......**错误!未定义书签。**
Figure s2 photo images and size distribution of spherical gelation after standing for 3d**错误!未定义书签。**
Figure s3 the stability testing of spherical gelation after standing for 1 year, the concentration of metal ions is 100 mM......**错误!未定义书签。**
Figure s4 optical images of spherical Cu/GG gelation with different size ...**错误!未定义书签。**
Table s1 elemental distribution of gelation sphere evaluated by EDS......5
Figure s5 SEM images of the cross-section of Cu/GG sphere after standing for 24h......5
Figure s6 the viscosity transformation affected by different additives at a shear rate of 200 1/s. The concentration of these additives is 10 mM. ......**错误!未定义书签。**
Figure s7 (a, b) SEM images of $Cu(OH)_2$ prepared by directly mixing Cu^{2+} (10 mM) and KOH solution (30 mM), and reacted at room temperature for 5 min......**错误!未定义书签。**
Figure s8 XRD pattern of $Cu(OH)_2$ and Cu/GG spheres. $Cu(OH)_2$ was prepared as the following procedure: 0.1208g $Cu(NO_3)_2{\cdot}3H_2O$ was added into KOH solution (1 M, 50 ml), after reacted for 2 min, the particles were obtained by centrifugation, then, the sample was washed with de-ionized water and freeze-dried. ......**错误!未定义书签。**
Figure s9 CV curves of HCPS-900 at various scanning rates......7
Figure s10 Nyquist plots of EIS of HPCS-900 (three-electrode system)......8
Fig. s11 cycling performance of HPCS-900 at a current density of 10 A/g......8

Supplementary materials

Methods

Synthesis of Cu/GG gel: 0.1208g $Cu(NO_3)_2·3H_2O$ was added into purified GG solution (0.5 wt%, 50 ml), after stirring for 30 min ensures the complete dissolution of salt, the mixture was poured into KOH solution (1 M, 50 ml), the as-obtained gel was named as Cu/GG gelation.

Synthesis of $Cu(OH)_2$/GG composite: Typically, 0.1208g $Cu(NO_3)_2·3H_2O$ was first weighed out and added into KOH solution (1 M, 50 ml), after standing for 30 min with stirring at temperature, the mixture was poured into purified GG solution (0.5 wt%, 50ml), and a gel consisted of $Cu(OH)_2$ and GG formed immediately.

Synthesis of CuO/GG composite: 0.1208g $Cu(NO_3)_2·3H_2O$ was added into KOH solution (1 M, 50 ml), then the mixture was transferred into a autoclave with Teflon lining, then it was treated at 100 °C for 12 h, After that, the solution was poured into purified GG solution (0.5 wt%, 50 ml), the final mixture was labelled as CuO/GG composite.

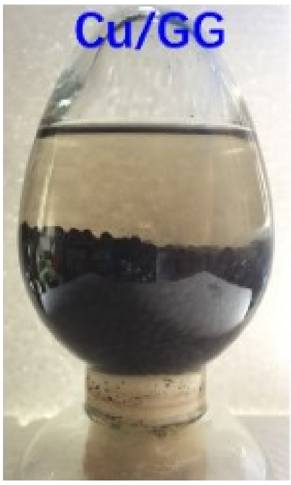

Figure s1 the stability testing of spherical hydrogel after standing for 1 year, the concentration of metal ions is 100 mM.

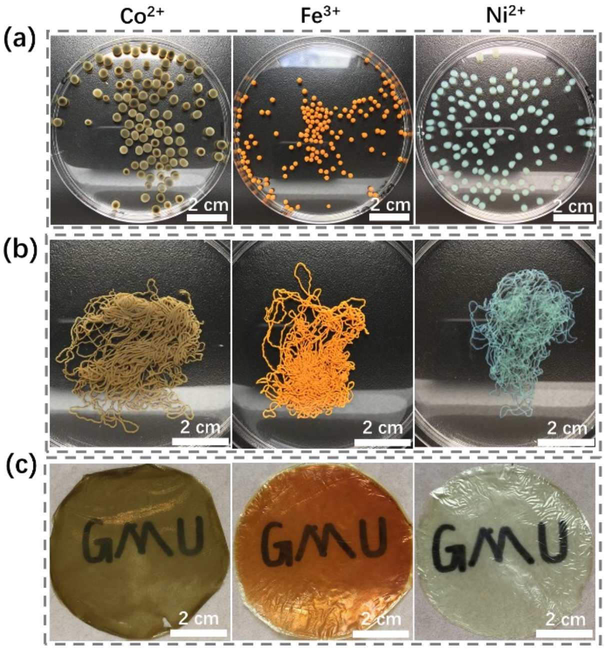

Figure s2 Co/GG, Fe/GG and Ni/GG hydrogel beads (a); fiber (b) and membrane (c)

Table s1 elemental distribution of hydrogel sphere evaluated by EDS

	Elements (wt%)	C	K	Cu	O
10s	Surface	26.56	24.04	13.53	35.87
	Cross-section	31.06	16.27	14.94	37.73
24h	Surface	36.13	2.80	12.54	32.75
	Cross-section	35.80	1.92	13.93	34.09

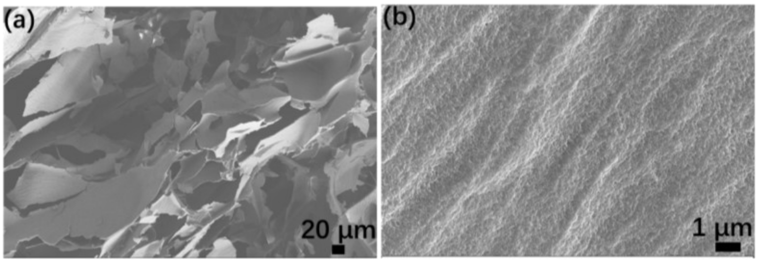

Figure s3 SEM images of the cross-section of Cu/GG sphere after standing for 24h

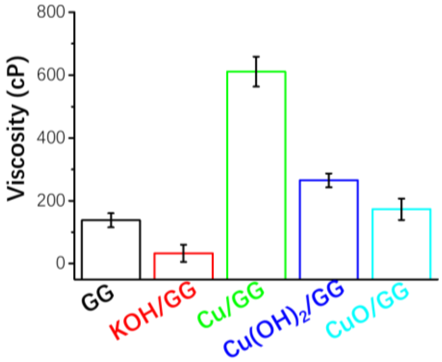

Figure s4 the viscosity transformation affected by different additives at a shear rate of 200 1/s. The concentration of these additives is 10 mM.

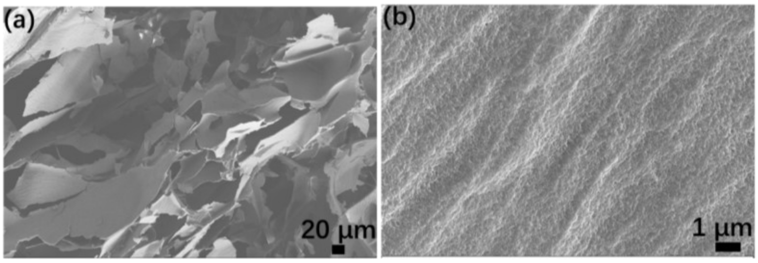

Figure s5 SEM images of the cross-section of Cu/GG sphere after standing for 24h

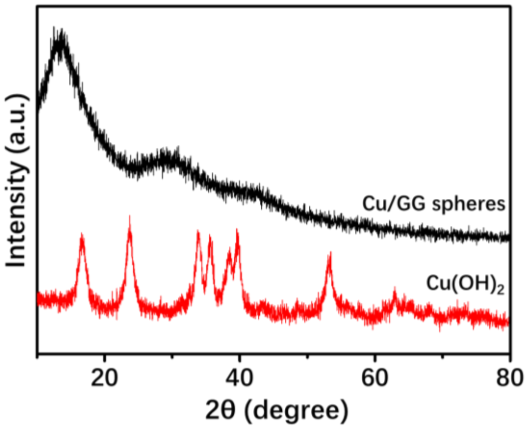

Figure s6 XRD patterns of $Cu(OH)_2$ and Cu/GG spheres. $Cu(OH)_2$ was prepared as the following procedure: 0.1208g $Cu(NO_3)_2{\cdot}3H_2O$ was added into KOH solution (1 M, 50 ml), after reacted for 2 min, the particles were obtained by centrifugation, then, the sample was washed with de-ionized water and freeze-dried.

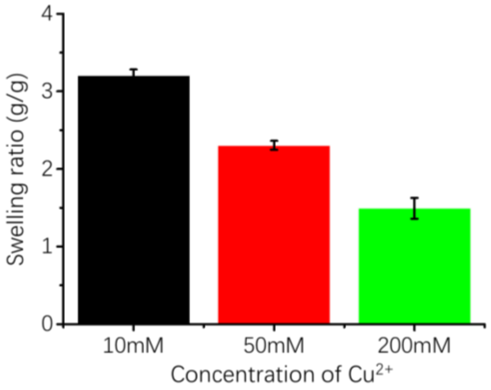

Figure s7 effect of the concentration of Cu^{2+} on the swelling ratio of Cu/GG hydrogel

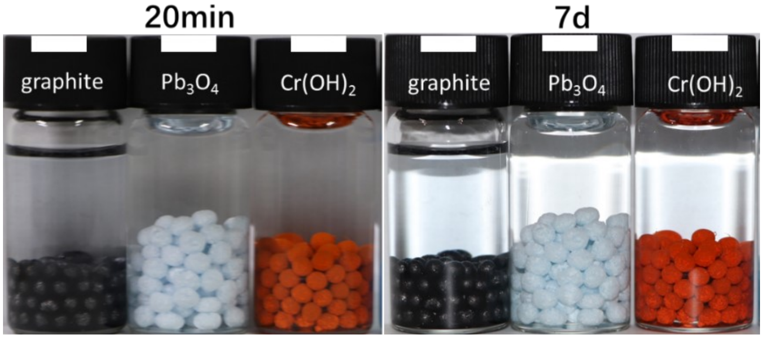

Figure s8 the encapsulation capacity and stability testing of Cu/GG hydrogel. The mole ratio of pollutant/Cu^{2+} is 200.

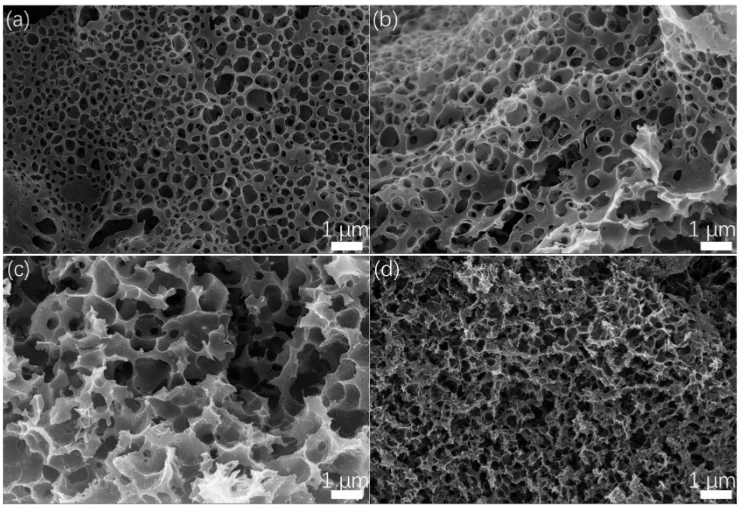

Figure 9 SEM images of HPCS samples prepared under different carbonization temperature

Table s2 pore structure analysis of the samples

Samples	S_{BET} (m^2g^{-1})	$S_{t\text{-}plot}$ (m^2g^{-1})	$S_{t\text{-}plot}$ /S_{BET}	$V_{t\text{-}plot}$ (m^3g^{-1})
**HPCS-400**	440	334	0.75	0.17
**HPCS-600**	690	611	0.88	0.32
**HPCS-800**	1280	930	0.79	0.48
**HPCS-900**	1524	660	0.44	0.33

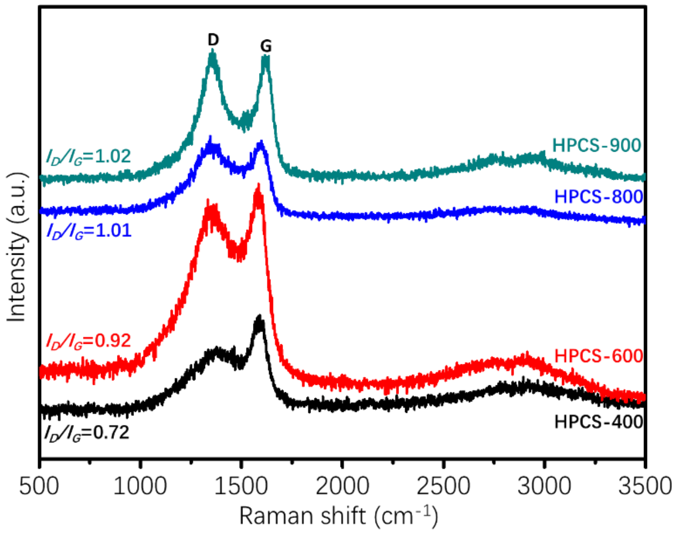

Figure 10 Raman spectra of the samples prepared at different carbonization temperature

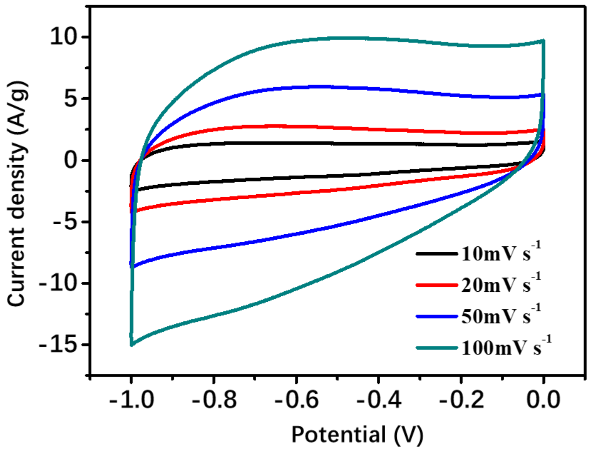

Figure s11 CV curves of HCPS-900 at various scanning rates

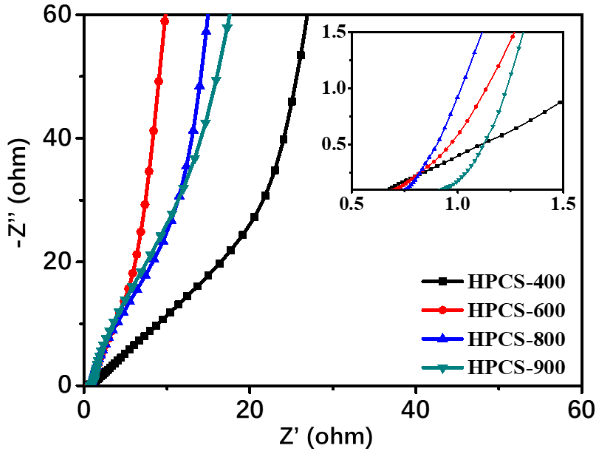

Figure s12 Nyquist plots of EIS of HPCS-900 (three-electrode system)

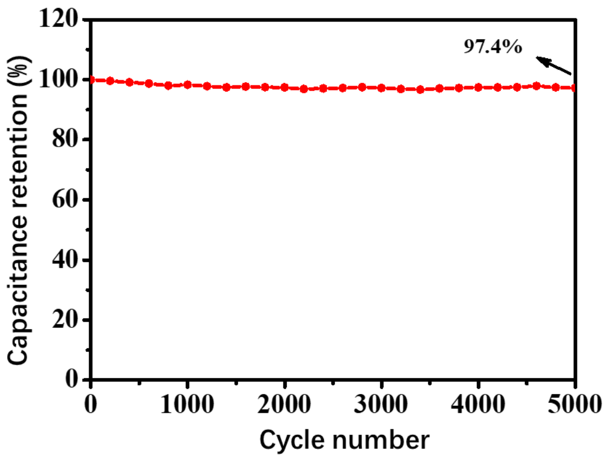

Fig. s13 cycling performance of HPCS-900 at a current density of 10 A/g
